# Role-players in abortion decision-making in the Accra Metropolis, Ghana

**DOI:** 10.1186/1742-4755-11-70

**Published:** 2014-09-16

**Authors:** Akwasi Kumi-Kyereme, Fred Yao Gbagbo, Joshua Amo-Adjei

**Affiliations:** Department of Population and Health, Faculty of Social Sciences, University of Cape Coast, Cape Coast, Ghana; Marie-Stopes International, Accra, Ghana

**Keywords:** Abortion, Role-players, Decision-making, Ghana

## Abstract

**Background:**

Making the final decision to terminate a pregnancy can be influenced by different circumstances involving various individuals. This paper describes the key players involved in the decision-making process regarding abortions among women who elected to undergo an induced abortion in a cosmopolitan urban setting in Ghana.

**Methods:**

A retrospective cross-sectional mixed method study was conducted between January and December 2011. A total of 401 women with records in abortion logbooks were selected for an interviewer-administered questionnaire and an in-depth interview. Descriptive and multinomial logistic regression analyses were used to assess the quantitative data, and a thematic analysis was applied to the qualitative data.

**Results:**

The findings of the study reveal that pregnant individuals, mothers of abortion-seekers, male partners, and “Others” (for example, friends, employers) were instrumental in making a decision to terminate unplanned/unwanted pregnancies. Several key factors influenced the decision-making processes, including aversion from the men responsible for the pregnancy, concerns about abnormalities/deformities in future births due to unprofessionally conducted abortions, and economic considerations.

**Conclusion:**

A number of individuals, such as friends, mothers, and male partners, influence the decision-making process regarding abortion among the participants of the study. Various targeted messages are needed for the various participants in the decision.

## Introduction

Induced abortion is permitted in Ghana for cases in which a woman’s life is at risk, when the procedure would preserve her physical and mental health, and on grounds of rape or incest. Despite the fact that induced abortion for any reason other than those mentioned above is illegal, prosecution of illegal abortion seekers, providers and role-players rarely occurs in the country. Furthermore, notwithstanding the public polemics on the morality of abortion, a large number of women undertake abortions around the world [1]. There were approximately 210 million pregnancies in 2008, with one out of every 10 ending in an unsafe abortion. An estimated 21.6 million unsafe abortions took place worldwide in 2008, an increase from 19.7 million in 2003. Almost all of those procedures occurred in developing countries, which translates into 14 unsafe abortions per 1000 women aged 15–44 years old. In Africa and Latin America, approximately 30 unsafe abortions occurred per 1000 women aged 15–44 years, although the range of estimates for Africa is wide [2]. In Ghana, induced abortion is reported to be the second highest cause of maternal mortality, regardless of the fact that it is generally legal [3].

Annually, approximately 205 million women worldwide become pregnant, and nearly one in five (40–50 million) choose to terminate the pregnancy for various reasons. Nearly half of all terminated pregnancies are estimated to be unsafe, and more than 80% occur in developing countries [1, 4].

The social, cultural and religious ambivalences [4] associated with induced abortion often complicate the decision-making process, making the decision to terminate an unwanted/unplanned pregnancy very difficult [5]. Although the decision to have an abortion is largely personal, it can also be influenced by political, economic and social factors [6]. These additional influences lead to many questions and emotions, which make the need for reliable and accurate information important. As a way of dealing with the nuances that surround abortion, some women tend to solicit the approval/consent of other people to help make the decision and to have the decision validated and implemented [7]. In other instances, women may be compelled through “orders” to abort, or by circumstances such as the father denying responsibility for the pregnancy [3].

Studies on the involvement of the individual and the involvement of “others” in the abortion decision-making process have reported multiple reasons for the ultimate decision, as well as varying attitudes of health service providers regarding the decision-making process [3, 8, 9]. At the personal level, evidence [10] has indicated that unwanted pregnancies can be considered a loss of personal control and that choosing abortion presents a means of restoring control, status, and normality. Kirkman et al. [11] noted that a woman’s decision-making regarding abortion is shaped by personal needs/concerns, the interests of the potential child, existing child/children, sexual partner and the extended family. Regarding the needs of the woman, feelings of not being ready and/or being too young for the commitment of motherhood have also been reported [11]. Additionally, instability of relationships, problems with previous male partners, and “new” relationships are some of the partner-related reasons for considering an abortion; abortion was favoured over raising a ‘fatherless’ child. Concerns about social prohibitions on pre-marital sex could push some women to abort pregnancies that occur out of formal or socially sanctioned unions. Thus, women would prefer abortion over “advertising” their sexual activities in highly conservative communities [3, 8, 9].

Hull and Hoffer [12] reported that the difficulties associated with abortion decision-making make the influence of various role-players very critical. Furthermore, these role-players have evolved from sympathetic midwives to the general range of health workers under the guise of protecting women from poorly trained abortion service providers [12]. A woman’s desire to safely terminate unwanted pregnancy and the professional responsibility of health workers have been cited as a basis for the need for collaboration with health workers to induce abortion, but it has also been noted that misinformation and the desperation of some women seeking abortion have resulted in women relying on the counsel of non-professional or unlicensed service providers, male partners, parents and friends [13].

In settings such as sub-Saharan Africa, where male chauvinism and gerontocratic tendencies are high, women who experience undesirable reproductive health outcomes must deal with potentially more nuanced decision-making processes than in contexts where these tendencies are low [14–17]. The complexities can be compounded among women who lack emotional, social and economic support, making autonomous abortion decision-making less probable [2].

This study examines the key influences in abortion decision-making in Ghana, relying on the views of women who underwent abortion between January and December 2010 in the Accra Metropolis. The study is important for both scholarship and advocacy. Regarding its significance for academic work, this study builds on one previous [3] study that has investigated the role of male partners, women and healthcare providers in Ghana. Effective advocacy also depends on evidence [11], and findings from this study are expected to contribute to the cases that would be built by advocates on abortion.

## Methods

### Study setting

This study was conducted in the Accra Metropolis, which has an estimated population of 2,242,505, of which 841,533 are within reproductive age [18]. As of 2010, there were 481 health facilities in the metropolis, consisting of four government hospitals, seven polyclinics, four quasi-government hospitals, 49 private hospitals, 270 clinics, 39 company clinics, 79 private maternity homes and 29 NGO/Mission hospitals. In 2008, approximately 17% (range: 8.1%-22.4%) of women (15–49 years) throughout the country reported a terminated pregnancy in the preceding five years. Disaggregated by region, 22.4% of women in the Greater Accra Region, where this study was conducted, had undergone an abortion [19], making it the region with the highest incidence of abortion in the country. In 2006, the Ghana Health Service, in collaboration with a consortium of five multinational organizations (EngenderHealth, Ipas, Marie Stopes International, Population Council, and the Willows Foundation) initiated the Reducing Maternal Mortality and Morbidity (R3M) project in the Greater Accra, Eastern and Ashanti regions following initial piloting in 17 districts in 2007. The R3M provides financial and technical assistance to enable the government to significantly expand access to modern family planning (FP) and comprehensive abortion care (CAC). The Accra Metropolis, a municipality within the Greater Accra that was involved in the pilot project, was purposively selected because of its cosmopolitan nature. Three accredited R3M health facilities, consisting of one private-not-for profit and two publicly owned and managed, were purposively selected. These were Marie Stopes International Ghana, BlueStar HealthCare Network, La and Ridge Hospitals. They were selected because of the high number of abortion cases registered in the metropolis in addition to the fact that they were the largest health facilities accredited to perform abortions under the R3M project. This context provides ‘friendly’ conditions for a safe abortion.

### Research design

This study employed a retrospective cross-sectional approach. Both qualitative and quantitative data were collected. This was done to complement the strengths and mitigate the limitations of the respective paradigms by using both questionnaires and in-depth interview (IDI) guides [20]. Neither the quantitative nor the qualitative data were given more weight [21], and the findings are presented concurrently. The questionnaire [22] and IDI guides [23, 24] were adapted from prior studies on abortion in Ghana. The questionnaire was structured around induced abortion decision-making processes, key individuals whose counsel was sought in the decision-making process, factors influencing choice of place for abortion and familiarity with the policy and legal frameworks, which influence induced abortion procedures in Ghana. The IDI guide, which was unstructured, focused on the following: pregnancy characteristics (e.g., person responsible), decision on whether to terminate, place of abortion, and choice of method, among others. Both questionnaires and interview guides were available in English but were translated into local languages for respondents who could not speak English. On average, each interview lasted approximately 60 min.

At the first stage of the sampling, a purposive method was used to select three abortion service providers accredited by R3M. The total number of women who had undergone an abortion between January and December 2010 was collated, resulting in an overall total of 9,494. This served as the sampling frame for the study, and individual women with a record of abortion served as the unit of analysis. With this, a sample size of 370 was drawn based on OpenEpi [25]. An additional 10% upward adjustment was made to correct for non-response. The sample was distributed among the facilities based on population (share of abortion cases) proportional to size (PPS). Thus, the proportions of the 9,494 women were allocated as follows: Marie Stopes (61), BlueStar (217), Ridge (53) and La (39) hospitals. Respondents were then selected randomly to respond to the interviewer-administered questionnaires. Another 35 respondents consisting of five previously married women, 10 unmarried women and 20 married women were selected to further explore the reasons for involving other individuals in abortion decision-making. Ten trained nurses served as research assistants. The fieldwork was conducted between June and December 2011. The Ghana Health Service Ethics Review Committee reviewed and approved the study. All research subjects provided verbal and written informed consent.

### Data analysis

#### Quantitative data

Four main groups of role-players were derived from the data: abortion seekers (self/personal autonomy), parents/mothers of abortion seekers, male partners, and less frequently mentioned role-players categorized as “Others”. Descriptive statistics with corresponding Chi-Square values were derived, followed by multinomial regression. Multinomial regression was used for inferential analysis because the dependent variable (role-players) has more than two outcomes, which makes it the most appropriate statistical tool for isolating the independent effects of the various categories of the background factors of abortion-seekers in abortion decision-making. The background factors captured in the quantitative data were occupation, age, marital status, religion, ethnicity, parity and number of previous abortions. These variables were entered concurrently because there was no intention to determine the impact of any single explanatory factor on the role-players.

### Qualitative analysis

The qualitative data were analysed inductively by identifying main themes. FYG first undertook preliminary coding of the data. AKK and JAA independently reviewed all codes, followed by identification, discussion and resolution of the inconsistencies in the themes by all of the authors. All three authors had to agree upon a particular theme before it was included in the codebook. Finally, a colleague with expertise in qualitative analysis reviewed our codes, comparing these to the transcripts, field notes and tape recordings. STATA version 12 (College Station, Texas 77845 USA) was used to analyse the quantitative data, while the qualitative data were analysed manually.

## Results

Overall, 32.67% (*n* = 131) of the respondents did not seek approval from anyone before receiving an abortion; 54.36% (*n* = 218) required their partner’s approval; 8.23% (*n* = 33) consulted with their mother for the decision; and the remaining 4.74% (*n* = 19) made the abortion decision with role-players categorized as “Others”, which includes friends, siblings, aunts/uncles, employers and mothers-in-law.

In this section, we present associations between background characteristics of abortion-seekers and role-players in abortion decision-making, which are presented in Table 1. The results indicate that women with secondary and higher education reported a higher level of involvement of their male partners in abortion decision-making than those with lower forms of education. Additionally, students/apprentices would rather involve their mothers in abortion decision-making (≈66%) than seeking the consent of male partners, others or deciding alone. Never married respondents also consulted their mothers (≈72%) more than they did a male partner (≈46%), alone (≈50%) or with others (≈58%). Women with zero parity were likely to decide with their mothers than male partners, solely or with others. Age of women, occupation, number of previous abortions and the number of living children showed significant associations with role-players in abortion decision-making.

**Table 1:** **Background characteristics of respondents and role-players in abortion decision-making in the Accra Metropolis, Ghana**

	Role-player					
	Personal	Sexual partner	Participant’s mother	Other	Total	N
**Knowledge of abortion law (** ***χ*** ^**2**^ **= 9.3528;** ***p =*** **0.155)**						
Abortion is legal	40.5	45.9	48.5	21.1	43.1	173
Abortion is illegal	14.5	19.3	9.1	21.1	17	68
Don’t know	45	34.9	42.4	57.9	39.9	160
**Educational level (** ***χ*** ^**2**^ **= 9.5561** ***p*** **= 0.145)**						
None/Primary	24.4	15.6	18.2	31.6	19.5	78
Middle/JSS	30.5	26.1	33.3	15.8	27.7	111
Secondary and Higher	45	58.3	48.5	52.6	52.9	212
**Ethnicity (** ***χ*** ^**2**^ ** = 7.7461;** ***p*** **= 0.560)**						
Akan	42.4	40.6	28.6	33.3	39.9	132
Ewe	17.0	20.0	32.1	20.0	19.9	66
Ga/Dangbme	28.0	21.2	28.6	33.3	24.8	82
Mole-Dagbani	12.7	18.2	10.7	13.3	15.4	51
**Religion (** ***χ*** ^**2**^ **= 12.2573;** ***p*** **= 0.056)**						
Christian	88.5	76.2	78.8	94.7	81.3	326
Moslem	11.4	22.0	18.2	5.3	17.5	70
Others	-	1.8	3.0	-	1.3	5
**Age group (** ***χ*** ^**2**^ **= 31.1048;** ***p*** **= 0.002)**						
15-19	14.6	7.4	36.4	11.1	12.3	49
20-24	30	34.6	39.4	38.9	33.7	134
25-29	28.5	30	18.2	16.7	27.9	111
30-34	15.4	18.9	3	16.7	16.3	65
35+	11.5	9.2	3	16.7	9.8	39
**Occupation (** ***χ*** ^**2**^ **= 38.6934;** ***p*** **= 0.000)**						
Unemployed	9.3	12.5	3.1	26.3	11.4	45
Self employed	50.4	32.9	25	21.1	37.4	148
Student/Apprentice	31	31.5	65.6	26.3	33.8	134
Other	9.3	23.1	6.2	26.3	17.4	69
**Marital status (** ***χ*** ^**2**^ **= 9.8489;** ***p*** **= 0.131)**						
Never married	49.6	46.3	71.9	57.9	50	199
Married/In union	40.5	45.4	25	42.1	42	167
Formerly married	9.9	8.3	3.1	0	8	32
**Number of previous abortions (** ***χ*** ^**2**^ **= 20.2018;** ***p*** **= 0.017)**						
None	30.4	17.9	35.5	22.2	23.4	88
One	41.7	60.4	41.9	61.1	53.2	200
Two	17.4	17.5	9.7	11.1	16.5	62
Three	10.4	4.2	12.9	5.6	6.9	26
**Number of living children (** ***χ*** ^**2**^ **= 20.9092;** ***p*** **= 0.052)**						
None	48.9	49.5	84.4	68.4	53	212
One	23.7	19.3	9.4	10.5	19.5	78
Two	16.8	14.7	3.1	10.5	14.2	57
Three	6.1	11	0	5.3	8.2	33
Four+	4.6	5.5	3.1	5.3	5	20

The results of further analysis using multinomial logistic regression are shown in Table 2 with the “Others” category as the reference. Respondents who indicated that abortion was illegal, as well as those who did not know the legal status of abortion reported decreased odds of making personal decisions regarding abortion vs. the “others” group. Increasing age also showed a negative relationship with personal autonomy in abortion decisions, holding “others” constant. The number of previous abortions was negatively related to personal autonomy; thus, having at least one previous abortion decreased the odds of making decisions about abortion alone compared to those who involved “others” such as friends, siblings, aunts, etc. On the other hand, education, occupation and the number of living children had a positive effect on personal autonomy compared to women who collaborated with “Others”. Evidence from the IDIs revealed that respondents appeared to have a great deal of personal autonomy, and this appeared to have been partially underscored by their socioeconomic status. This was confirmed by one of the respondents:

**Table 2:** **Multinomial logistic regression results on role-players in abortion decision-making by background characteristics of respondents**

	***Personal/self***		***Male partner***		***Participant’s nother***	
	Coefficient	95% CI	Coefficient	95% CI	Coefficient	95% CI
**Knowledge of abortion**						
Abortion is not legal	0	[0,0]	0	[0,0]	0	[0,0]
Abortion is illegal	−1.013	[−3.070,1.044]	−0.884	[−2.886,1.118]	−1.683^*^	[−4.053,0.686]
Don’t know	−1.030	[−2.672,0.612]	−1.248	[−2.856,0.360]	−1.522	[−3.418,0.374]
**Level of education**						
None/Primary	0	[0,0]	0	[0,0]	0	[0,0]
Middle/JSS	0.649	[−1.506,2.803]	1.097	[−1.034,3.229]	1.024	[−1.449,3.497]
Secondary and Higher	0.561	[−1.247,2.369]	0.947	[−0.826,2.719]	0.430	[−1.769,2.629]
**Ethnicity**						
Akan	0	[0,0]	0	[0,0]		
Ewe	−0.240	[−2.161,1.682]	−0.169	[−2.048,1.710]	0	[0,0]
Ga/Dangbme	−0.242	[−1.830,1.346]	−0.294	[−1.844,1.255]	0.273	[−1.937,2.483]
Mole-Dagbani	−1.312	[−4.236,1.613]	−1.279	[−4.154,1.596]	0.923	[−0.994,2.840]
**Religion**						
Christian						
Moslem	1.453	[−1.810,4.716]	2.262	[−0.952,5.476]	1.879	[−1.721,5.479]
Others	−0.589	[−15911.6,15910.4]	17.01	[−14202.2,14236.2]	18.37	[−14200.8,14237.6]
**Age**						
15-19	0	[0,0]	0	[0,0]	0	[0,0]
20-24	−0.308	[−2.509,1.893]	0.497	[−1.683,2.676]	−0.718	[−3.068,1.632]
25-29	1.362	[−1.583,4.306]	1.736	[−1.187,4.659]	0.386	[−2.851,3.623]
30-34	−1.085	[−4.019,1.849]	−0.647	[−3.514,2.220]	−17.01	[−2853.4,2819.4]
35+	−1.371	[−4.240,1.498]	−1.015	[−3.847,1.817]	−2.568	[−6.618,1.483]
**Occupation**						
Unemployed	0	[0,0]	0	[0,0]	0	[0,0]
Self-employed	1.973	[−0.0726,4.018]	1.366	[−0.598,3.329]	1.564	[−1.407,4.535]
Student/Apprentice	1.893	[−0.133,3.919]	1.601	[−0.330,3.532]	2.769	[−0.0294,5.567]
Other	0.700	[−1.683,3.083]	1.408	[−0.821,3.637]	1.893	[−1.434,5.220]
**Marital status**						
Never married	0	[0,0]	0	[0,0]	0	[0,0]
Married/In-union	−0.924	[−2.482,0.635]	−0.608	[−2.128,0.911]	−1.144	[−3.066,0.779]
Formerly married	15.09	[−3811.3,3841.5]	15.15	[−3811.2,3841.5]	14.69	[−3811.7,3841.1]
**Number of previous abortions**						
None	0	[0,0]	0	[0,0]	0	[0,0]
One	−1.459	[−3.316,0.398]	−0.0657	[−1.922,1.791]	−0.864	[−2.954,1.226]
Two	−0.647	[−3.127,1.833]	0.310	[−2.162,2.783]	−0.716	[−3.681,2.249]
Three+	−0.836	[−4.110,2.438]	0.0392	[−3.232,3.311]	0.571	[−2.958,4.100]
**Number of living children**						
None	0	[0,0]	0	[0,0]	0	[0,0]
One	1.920	[−0.450,4.290]	1.594	[−0.743,3.931]	1.397	[−1.336,4.131]
Two	1.093	[−1.460,3.645]	1.181	[−1.320,3.683]	0.314	[−3.066,3.695]
Three	1.480	[−1.676,4.636]	2.151	[−0.899,5.201]	−12.50	[−3675.7,3650.7]
Four+	1.487	[−2.106,5.079]	1.107	[−2.408,4.622]	1.906	[−2.789,6.601]
Constant	1.779	[−0.922,4.479]	0.512	[−2.222,3.247]	0.00180	[−3.547,3.550]
*AIC*	669.7					
Log likelihood	−256.8					
Chi-squared	110.5					
*N*	299					

*p < 0.05, **p < 0.01, ***p < 0.001.

Reference category: “Others”.

*As an adult, I knew what was ok for me; hence, I did not need anybody’s consent to have an abortion. It was a personal decision and so I went for abortion when I made-up my mind the pregnancy was not needed at this time*. (25 years old nurse)

The trends in seeking the consent or approval of male sexual partners generally followed the pattern noted for personal autonomy, albeit some slight variations existed, as shown in Table 2. Respondents who knew that abortion was illegal and those who did not know the legal status of abortion reported lower odds of involving their male partners relative to “Others”. Occupation status, number of living children living and level of formal education led to increased odds of seeking the consent of male partners apropos of “Others”. The role of male partners in female reproductive health affairs is seemingly enormous. Male partners may either restrain or encourage their female partners to abort an unwanted pregnancy. Economic power is fundamental to this dynamic. It appeared that some respondents decided to abort their pregnancy because of the breadwinner financial role of the father. They appeared to be anxious of the possible implications of keeping a pregnancy in the case that a man indicated an unwillingness to support economically. One respondent asserted:

*I had no choice but to comply with my husband’s directive to have an abortion because he was the breadwinner of the family and was not ready to have another child at the time*. (32 years house wife)

Compared to the “Others”, a partnership with mothers in abortion decision-making was negative among those who knew that abortion was illegal and those who were not knowledgeable of the abortion law. Decreased odds of involving mothers related to “Others” are noted among various groupings of age. Married/in-union women, those with one and two previous abortions, and those women with two and three living children reported decreased odds of involving their mothers relative to “Others” in abortion decision-making. The motivations for respondents involving “Others” in the decision-making process varied: for some mothers, they simply did not like the man responsible. One respondent narrated:

*When I became pregnant, my mother was very disappointed in me not because of the pregnancy but because of the man with whom I was pregnant for. She did not support any relationship with the guy who got me pregnant. She therefore did everything possible to convince me to have the pregnancy terminated, so much so that I obliged to make her happy*. (21 year old teacher)

For other respondents, their mothers-in-law were brought into the decision-making process to aid in the final decision. The participation of mothers-in-law was occasionally to convince a woman to accept abortion as a means of saving the life of the woman. In other instances, it was to avoid bearing children with foetal abnormalities. One respondent shared her experience as follows:

*The decision to have an abortion was difficult for my husband and I since we had been expecting a pregnancy for six years. When I finally became pregnant, my doctor indicated that the child had gross foetal abnormalities and hence convinced my mother in-law to persuade us to have the pregnancy terminated, which we did*. (35 year old businesswoman)

## Discussion

Induced abortion, along with its associated health challenges, continues to remain a public health concern in low-income countries, even where the practice is legal. Whether conducted legally or illegally, decisions on abortion are complicated, and this sometimes necessitates the involvement of invited or uninvited role-players. This paper sought to highlight some of these role-players and the influences on and of their specific roles. The findings of the study reveal that pregnant individuals, mothers of abortion seekers, male partners, and “Others” such as friends and employers were instrumental in making a decision to terminate unplanned/unwanted pregnancies. These individuals/stakeholders were motivated by the need to maintain ownership of their bodies, the need to uphold individual rights, economic reasons, avoidance of future foetal abnormalities and the maternal view of ideal partners for their pregnant daughters.

A key strength of this study is that both survey-administered interviews and IDIs were conducted in communities, and the research staff and individual respondents determined the place of interview together. This is in contrast to most post-abortion studies in which data collection occurs in health facilities [3, 26–29] and carries a high risk of bias introduced by the health facility environment. Another strength of this study is the concurrent qualitative and quantitative methods, which ensured both breadth and depth and which is rarely the case in abortion research, to the best of our knowledge. Nevertheless, the fact that respondents were retrospectively selected presents potential risks of information selection and misclassification biases. The possibility that respondents lost track of certain important events does exist. The study was conducted between January and December 2011, while abortion events occurred between January and December 2010, suggesting that the minimum time frame between an abortion event and the study ranged between one month and 24 months. Additionally, the limited sample size challenges any claims of generalizability of the findings; the sample set precludes women who may have undergone an abortion in unlicensed facilities.

Partners’ consent influenced the majority of decisions regarding abortion. One of the seminal studies on influences of male partners on abortion decision-making was conducted by Browner [30]. Browner’s [30] research in Colombia revealed that women accepted abortion proposals from their male partners for fear of having children whose fathers have refused responsibilities. Similar findings have been reported from two Ghanaian tertiary hospitals [3]. Another important reason for male partners’ control on abortion decision-making is related to the women’s fears of being accused of infidelity [31]. In some countries (e.g., Turkey) where conservative norms are prevalent, women are required to provide partner’s consent [31]. Additionally, the precarious economic condition of a significant proportion of women in Africa makes them highly dependent on their male partners in financing abortion, and if they experience complications, they may delay care-seeking or may not be able to obtain abortion services at all without the approval of men [32]. However, in some contexts, women may ignore male partners’ approval following abuse [33]. Conversely, male partners’ role in women’s abortion decision-making should not be seen as adversarial. Of course, women are rational, and therefore, decisions regarding abortion might be weighed against the quality of support from the partner in keeping or terminating a pregnancy. Thus, if the perceived support is low, abortion will be a reasonable option [34], and as Byrnes [35] notes, expectations are principal in the decision-making process.

Mothers of the individuals involved in the study were found to have played crucial roles in aborting pregnancies. Young women’s reproductive health and pregnancy decision-making processes are shaped by familial and community expectations and norms. Views of mothers and parent figures have been reported to be key moderating factors in the decision-making on whether to maintain a pregnancy or not [36]. Where mothers are liberal about abortion, they may encourage their female children to opt for abortion, and this may be heightened by parental views on an ideal husband for their daughters. This idealistic view may be shaped by economic, religious, cultural/ethnic, educational attainment, and several other factors. Where perceived family ideals conflict with realities, mothers may turn to induce abortion to resolve such perceived “devastations”. In a study in Kenya [37], some parents, particularly mothers, warned their children not to get pregnant by poor boys/men. Ralph et al. [38] have demonstrated similar maternal pressures in their daughter’s abortion decision-making. The findings also corroborate Foster et al.’s [39] observation about the role of mothers in enhancing the confidence of their daughters in seeking abortion.

Some women also showed a certain level of autonomy, and abortion was considered as a means of expressing self-control. Women’s desire to seek induced abortion without consulting with any other person is informed by personal desires of taking charge of their lives after perceived slackness in making ‘responsible’ reproductive health choices. Thus, abortion may be considered an option to restoring personal dignity and identity [10]. Here, the agency role is critical. Thus, a woman faced with a complex decision of whether to terminate or keep an unwanted/unplanned pregnancy reflects on expectations and uncertainties in getting to the final destination of abortion or full-term pregnancy [33]. For instance, a woman who became pregnant through an extra-marital affair may fear that involving others in the decision-making process might lead to marital disruption and therefore might make the decision solely.

The narratives of the respondents reveal that unplanned pregnancies ultimately influenced abortion decision-making. This suggests the need for intensive campaigns for increased use of contraceptives, particularly in post-abortion family planning, given the high probability of repeated abortions among women with a history of abortion. To further enhance our understanding of abortion decision-making processes, male partners, mothers of abortion-seekers, abortion history, friends and other role-players should be targeted in future studies.

## Conclusion

Abortion decision-making remains a public health issue. In a liberal abortion context such as that of Ghana, it is important to identify role-players in the decision-making process regarding abortions. Because the decision to have an abortion is not always an expression of a pregnant woman’s sentiments alone, there is a need to provide culturally appropriate support systems for those who desire to have an abortion. This will ensure that unsafe abortion episodes are reduced to minimum levels.
